# Yougui Pills Attenuate Cartilage Degeneration via Activation of TGF-β/Smad Signaling in Chondrocyte of Osteoarthritic Mouse Model

**DOI:** 10.3389/fphar.2017.00611

**Published:** 2017-09-05

**Authors:** Lei Zhang, Ping-er Wang, Jun Ying, Xing Jin, Cheng Luo, Taotao Xu, Shibing Xu, Rui Dong, Luwei Xiao, Peijian Tong, Hongting Jin

**Affiliations:** ^1^Institute of Orthopaedics and Traumatology, The First Affiliated Hospital of Zhejiang Chinese Medical University Hangzhou, China; ^2^The First College of Clinical Medicine, Zhejiang Chinese Medical University Hangzhou, China; ^3^Department of Orthopaedics and Traumatology, Wangjiang Sub-District Community Health Service Center Hangzhou, China; ^4^Department of Orthopaedic Surgery, The First Affiliated Hospital of Zhejiang Chinese Medical University Hangzhou, China

**Keywords:** Yougui pills, osteoarthritis, conditional knockout, cartilage protective, TGF-β/Smad signaling

## Abstract

Yougui pills (YGPs) have been used for centuries in the treatment of Chinese patients with Kidney-Yang Deficiency Syndrome. Despite the fact that the efficiency of YGPs on treating osteoarthritis has been verified in clinic, the underlying mechanisms are not totally understood. The present study observes the therapeutic role of YGPs and mechanisms underlying its chondroprotective action in osteoarthritic cartilage. To evaluate the chondroprotective effects of YGPs, we examined the impact of orally administered YGPs in a model of destabilization of the medial meniscus (DMM). Male C57BL/6J mice were provided a daily treatment of YGPs and a DMM surgery was performed on the right knee. At 12 weeks post-surgery, the joints were harvested for tissue analyses, including histomorphometry, OARSI scoring, micro-CT and immunohistochemistry for COL-2, MMP-13 and pSMAD-2. We also performed the relative experiments mentioned above in mice with *Tgfbr2* conditional knockout (*TGF-βRII^Col2ER^* mice) in articular cartilage. To evaluate the safety of YGPs, hematology was determined in each group. Amelioration of cartilage degradation was observed in the YGPs group, with increases in cartilage area and thickness, proteoglycan matrix, and decreases in OARSI score at 12 weeks post surgery. In addition, reduced BV/TV and Tb. Th, and elevated Tb. Sp were observed in DMM-induced mice followed by YGPs treatment. Moreover, the preservation of cartilage correlated with reduced MMP-13, and elevated COL-2 and pSMAD-2 protein expressional levels were also revealed in DMM-induced mice treated with YGPs. Similarly, *TGF-βRII^Col2ER^* mice exhibited significant OA-like phenotype. However, no significant difference in cartilage structure was observed in *TGF-βRII^Col2ER^* mice after YGPs treatment. Interestingly, no obvious adverse effects were observed in mice from each group based on the hematologic analyses. These findings suggested that YGPs could inhibit cartilage degradation through enhancing TGF-β/Smad signaling activation, and be considered a good option for the treatment of osteoarthritis.

## Introduction

Osteoarthritis (OA), a disease of articular joints, is associated with significant morbidity, mortality, physical disability, and increased healthy care expenditures in middle age and elderly individuals (Helmick et al., 2008; Hochberg, 2012). An improved understanding of disease pathogenesis and advances in the investigation of biomarkers is facilitating much emphasis in the prevention and treatment of early OA (Glyn-Jones et al., 2015). Traditionally, a variety of treatment options for OA consists of pain management for early stage disease through medication. Pharmaceuticals, especially non-steroidal anti-inflammatory drugs (NSAIDs), already play an important part in symptom control (Hochberg et al., 2012). However, the long-term uses of these drugs may produce severe adverse effects. In addition, none has so far been approved by regulators, which require concurrent symptom amelioration and disease modification (Conaghan et al., 2011).

Traditional Chinese medicine (TCM) acts as the promising alternative medicine as a therapeutic option for OA (Li et al., 2017). According to the TCM theory, the primary pathogenesis of OA is insufficiency of liver and kidney (Chen et al., 2016). Based on the therapeutic principle, a classic TCM formula Yougui pills (YGPs) which is first recorded in *Jingyue quanshu* has been used to treat Kidney-Yang Deficiency Syndrome with a history of about 400 years in China. Previous study published in Chinese language showed that YGPs has chondroprotective effects on OA (Pan and Li, 2010). Although YGPs has been proved effective in OA treatment, the precise mechanism and drug action target is still poorly understood.

Cartilage degeneration is a remarkable feature of OA and it may be an important pathological event contributing to the development of OA. The characteristics of articular cartilage degradation include proteoglycans and collagen degeneration, chondrocyte clustering, fibrillation and osteophyte formation (Xu et al., 2003). However, adult mammal’s articular cartilage appears to show a poor ability of repairing itself throughout life (Lietman et al., 2002; Correa and Lietman, 2017). The transforming growth factor beta (TGF-β) signaling in cartilage which is responsible for regulating the synthesis and degradation of extracellular matrix (ECM) protein, for controlling the proliferation and differentiation of chondrocyte and for inhibiting it hypertrophy and maturation plays a crucial role in pathogenesis of OA (Blaney et al., 2007; Quintana et al., 2009; Cuellar and Reddi, 2015). Recently, related studies showed that inhibition of TGF-β signaling in cartilage induces articular cartilage degradation (Blaney et al., 2007; Shen et al., 2013; Wu et al., 2015). Therefore, pharmacological activation of TGF-β signaling has been proposed to maintain articular cartilage integrity during OA (Blaney et al., 2007).

The aim of the current study was the histological observation of the chondroprotective effect of YGPs on OA progression in the articular cartilage of wild type (WT) and *Tgfbr2* conditional knockout (cKO) mice. We hypothesized that the chondroprotective activity of YGPs on OA *in vivo* based on enhancing TGF-β/Smad signaling in articular cartilage.

## Materials and Methods

### Reagents

DNA extraction kit and Tamoxifen were purchased from Sigma–Aldrich (Sigma-Aldrich, St. Louis, MO, United States). 4% paraformaldehyde, 14% elhylene diamine tetraacetic acid (EDTA) and citrate buffer were purchased from Solarbio (Solarbio, Beijing, China). Normal goat serum, secondary biotinylated goat anti-mouse antibody and diaminobenzidine (DAB) solution were obtained from Invitrogen (Invitrogen, Frederick, MD, United States). COL-2, MMP-13 and pSMAD-2 primary antibodies were brought from Abcam (Abcam, Cambridge, MA, United States).

### Preparation of YGPs

Yougui pills were produced by Henan Wanxi Pharmaceutical Co. Ltd. (Batch number: Z41022170) Small amount of sample was deposited as voucher specimen in institute of orthopedics and traumatology, Zhejiang Chinese Medical University with voucher specimen No. GYS-2015-0016. YGPs comprise 10 kinds of herbal medicines (**Table 1**). The production process was based on the standards of Chinese Health and Family Planning Commission. Briefly, *Cervicornuscolla* was firstly melted with wine by heat and the other nine herbal medicines were ground to fine powder. Every 100 g powder was mixed with 65 g molten *Cervicornuscolla* and honey (Ji et al., 2016). The high performance liquid chromatography (HPLC) detection was performed to analysis of and the quality control of YGPs. The analysis procedure was conducted according to the previous reports (Lin et al., 2012). Ten of the major constituents including betaine, sweroside, loganin, hyperin, quercitrin, chlorogenic acid, songorine, fuziline, neoline and cinnamaldehyde were determined in our study.

**Table 1:** The compositions of Yougui pills (YGPs).

Chinese name	Botanical name	Latin name	Weight (g)	Parts used
Shu di huang	*Rehmannia glutinosa* (Gaertn). DC.	*Rehmanniae radix praeparata*	240	Root
Shao yao	*Dioscorea oppositifolia* L.	*Dioscoreaerhizoma*	120	Rootstock
Shan zhu yu	*Cornus officinalis* Sieb. EtZucc.	*Cornifructus*	90	Fruit
Gou qi zi	*Lyciumbarbarum* L.	*Lyciifructus*	120	Fruit
Lu jiao jiao	*Cervus elaphus* Linnaeus	*Cervicornuscolla*	120	Horn
Tu si zi	*Cuscuta chinensis* Lam.	*Cuscutae semen*	120	Fruit
Du zhong	*Eucommia ulmoides* Oliv.	*Eucommiae cortex*	120	Bark
Dang gui	*Angelica sinensis* (Oliv.) Diels	*Angelicaesinensis radix*	90	Root
Rou gui	*Cinnamomum cassia* (L.) J. PreslBark	*Cinnamomi cortex*	60	Bark
Fu zi	*Aconitum carmichaeli* Debx.	*Aconitilateralis radix preparata*	60	Root

### Animals

Male C57BL/6J mice (10 weeks old) were obtained from Shanghai Laboratory Animal Center of Chinese Academy of Sciences (Grade SPF II, SCXK 2012-0002). *Col2-CreER* and *TGF-βRII^fx/fx^* mice (background: C57BL/6J) were donated from Rush University Medical Center (Chicago, IL, United States). In order to specifically knock out *Tgfbr2* gene in articular cartilage, *TGF-βRII^fx/fx^* mice were cross with *Col2-CreER* mice to generate *Col2-CreER; TGF-βRII^fx/fx^* mice (*TGF-βRII^Col2ER^* mice) (Shen et al., 2013). Genotyping was performed by polymerase chain reaction (PCR) using a DNA extraction kit tail biopsy tissues (Supplementary Figure S1). PCR primer sequences as follows: primer sequences of *Cre*, upper primer 5′-ATTGCTGTCACTTGGTCGTGGC-3′ and lower primer 5′-GAAAATGCTTCTGTCCGTTTGC-3′ (200-base-pair PCR product); primer sequences of *Tgfbr2* loxP, upper primer 5′-TAAACAAGGTCCGGAGCCCA-3′ and lower primer 5′-ACTTCTGCAAGAGGTCCCCT-3′ (Wild-type, 420-base-pair PCR product; homozygotic type, 540-base-pair PCR product). *TGF-βRII^fx/fx^* littermates were used as controls (Wang et al., 2013; Shen et al., 2014; Chen et al., 2017). After identification of genotypes, *TGF-βRII^Col2ER^* mice were induced by tamoxifen (1 mg/10 g body weight/day, i.p.) for five successive days at age of 2-week-old (Shen et al., 2013). All mice were housed in a temperature (23 ± 2°C) and humidity (40 ± 5%) controlled room, exposed to a controlled 12 h cycle of light and darkness with a solid rodent chow and water *ad libitum*. All studies were approved by the Committee on the Ethics of Animal Experiments of Zhejiang Chinese Medical University.

### Surgical Preparation

Knee OA model was induced to C57 mice at age of 10-week-old by destabilization of the medial meniscus (DMM) as previous report (Iijima et al., 2014). Briefly, DMM surgery was performed on the right hind limbs of WT mice as follows: (1) After a 3 mm longitudinal incision was made on the medial part of the knee under anesthesia, blunt dissection of the knee extensor muscles and patellar ligament was performed to expose the medial meniscotibial ligament (MMTL); (2) The MMTL was transected to give DMM; (3) The medial joint capsule was sutured, and the skin was closed. The sham surgery was also performed by a similar surgical approach without manipulating the joint tissue in WT mice.

### Experimental Protocol

All mice were randomly divided into six groups: the sham group, the model group, the model YGPs group, the *TGF-βRII^fx/fx^* group, the *TGF-βRII^Col2ER^* group and the *TGF-βRII^Col2ER^* + YGPs group (*n* = 10 in each group). YGPs were orally administered to both WT and *TGF-βRII^Col2ER^* mice once a day after DMM surgery and tamoxifen inducement respectively for 12 consecutive weeks with dose of 3.5 g/kg body weight. The dose administered was determined according to the following formula: *D*_M_ (dose per kg body weight) = *D*_H_ × *R* × (*W*_H_/*W*_M_), as detailed in The Methodology of Pharmacological Experiment. *D*_M_ and *D*_H_ are doses for mice and humans, and *W*_M_ and *W*_H_ are body weights of mice and humans, respectively. *R* is the coefficient of 0.0026 for human mouse equivalent dosage conversion (Xu et al., 2002). The mice in sham and *TGF-βRII^fx/fx^* group were given an equal dosage of physiologic saline. Knee joint samples from each group were harvested at the end of drug intervention period for the follow-up experiments.

### Assessment of Articular Cartilage Degradation

Knee joints of each group were successively fixed in 4% paraformaldehyde for 3 days and decalcified with 14% EDTA solution for 14 days. The samples were embedded in paraffin, and a series of histologic sections (3 μm) were stained with Alcian Blue Hematoxylin/Orange G and Toluidine Blue. The image of sections was collected under a light microscope (Axio Scope A1, ZEISS, Germany). Next, Histomorphometric analysis was performed using OsteoMeasure software (OsteoMetrics, Inc., Atlanta, GA, United States) to calculate and determine the area and thickness of cartilage. Moreover, according to Osteoarthritis Research Society International (OARSI) recommendations (Glasson et al., 2010), cartilage degeneration was scored by two blinded observers. The summed score for the knee joint (determined by summing the scores for the femoral condyle and tibial plateau) was used to evaluate the extent of cartilage degradation.

### Micro-CT Analysis

The representative knee joint images of mice were obtained from micro-CT equipment (Skyscan 1176, Bruker microCT N.V., Kontich, Belgium). The pictures were taken with a resolution of 4000 × 2672 pixels and an isotropic voxel size of 9 μm. In addition to the visual assessment of structural pictures, quantitative morphometry indexes were determined from micro-tomographic data based on the 3-D morphometry (Hildebrand et al., 1999). The region of interest was identified between the proximal tibia growth plate and tibial plateau. And the following indexes were evaluated subsequently: (1) bone volume fraction (BV/TV, %); (2) average trabecular thickness (Tb. Th, mm); (3) average trabecular separation (Tb. Sp, mm).

### Immunohistochemistry

We also performed immunohistochemistry to analyze the expressions of type II collagen (COL-2), matrix metalloproteinase 13 (MMP-13) and phosphorylated protein mothers against decapentaplegic homolog 2 (pSMAD-2) in articular cartilage. The paraffin sections were heated at 60°C overnight, then deparaffinized and rehydrated. Next, the sections were incubated with 3% hydrogen peroxide to block endogenous peroxidase activity for 20 min. the sections were incubated in 0.1 mol/L citrate buffer (Zhongshan jinqiao, Beijing, China) at 95°C with water bath for 20 min as antigen retrieval. Immunohistochemistry analysis was achieved using primary antibodies of COL-2 (diluted 1:1000), MMP-13 (diluted 1:100) and pSMAD-2 (diluted 1:100) at 4°C overnight. After washing, the sections were incubated with the HRP-conjugated secondary antibody at room temperature for 30 min. After incubated with DAB, counterstained with hematoxylin for 2 s, positive staining of sections was observed under a light microscope (Axio Scope A1, ZEISS, Germany).

### Routine Blood Test

Routine blood test was performed at the end of the experiment. All mice were fasted but allowed to water freely for 12 h prior to collecting the blood sample. The blood samples were collected from the murine orbit, followed by the centrifugation in heparinized tubes. Hematologic indexes included white blood cell (WBC) count, red blood cell (RBC) count and platelet (PLT) count.

### Statistical Analysis

All the data were expressed as mean ± SD. One-way analysis of variance followed by Dunnett’s test when appropriate. *P* < 0.05 was considered to be statistically significant. The statistical analysis was performed using SPSS 18.0 software (SPSS, Chicago, IL, United States).

## Results

### YGPs Inhibit DMM-Induced OA-Like Phenotype

**FIGURE 1:**
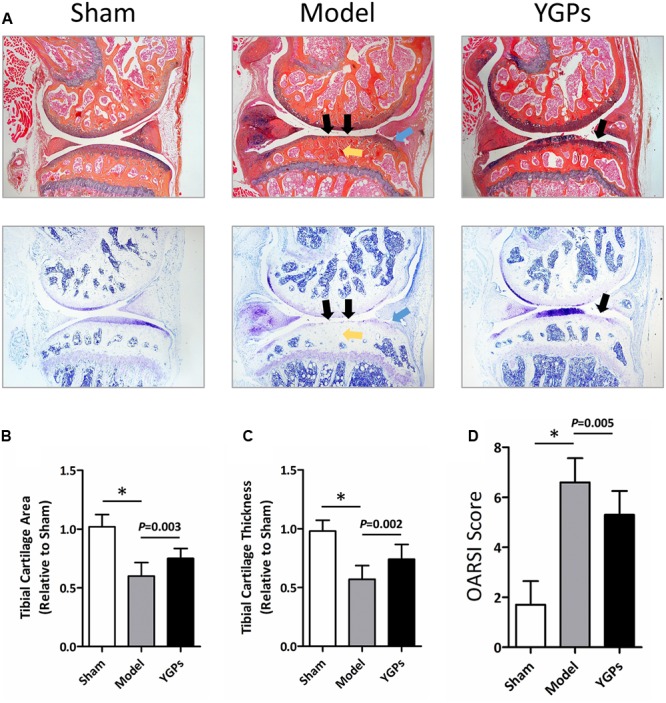
Yougui pills (YGPs) protect against cartilage loss in DMM-induced mice. **(A)** Histological knee joint sections (50×) stained using Alcian Blue Hematoxylin/Orange G and Toluidine Blue. Joint degenerations are labeled (black arrows: cartilage degradation, blue arrows: osteophyte formation, yellow arrows: subchondral sclerosis). Cartilage architecture was evaluated using the Osteomeasure System to detemine the tibial cartilage area **(B)** and tibial cartilage thickness **(C)** OARSI scoring of the sections analyzed by histomorphometry was also performed **(D)** Bars represent Mean ± SD (*n* = 10). ^∗^*P* < 0.001.

To evaluate cartilage degradation induced by DMM surgery, Alcian Blue Hematoxylin/Orange G and Toluidine Blue stainings were subsequently performed. As shown in **Figure 1A**, no pathological findings of OA were observed in mice from sham group. In contrary, DMM-induced mice exhibited localized damage of articular cartilage, early osteophyte formation and increases in subchondral bone mass. By treated with YGPs, cartilage surface erosion and joints degradation were noticeably decreased. Histomorphometric analysis showed a significant reduction in tibial cartilage area and thickness in OA model mice (both, *P* < 0.001). Likewise, prominent increase of tibial cartilage area (**Figure 1B**) and thickness (**Figure 1C**) also occurred after YGPs treatment compared with model group (*P* = 0.003; *P* = 0.002). Moreover, we analyzed the data using the histologic scoring system recommended by OARSI (Glasson et al., 2010), and we found that DMM-induced mice had significantly higher scores compared to sham group (*P* < 0.001), with significantly downregulated OARSI scores after treatment with YGPs (*P* = 0.005) (**Figure 1D**).

**FIGURE 2:**
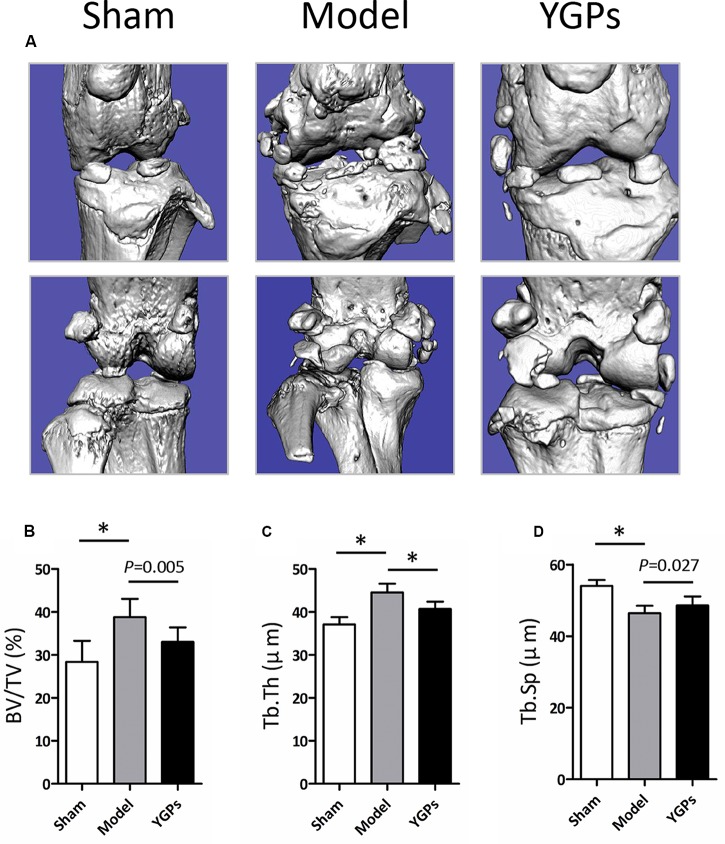
Changes in bone structure in DMM-induced mice. **(A)** Representative micro-CT images. Quantification of the **(B)** BV/TV, **(C)** Tb. Th and **(D)** Tb. Sp by static histomorphometry. Bars represent Mean ± SD (*n* = 10). ^∗^*P* < 0.001.

To assess the microarchitecture of the bone, the three-dimensional image was carried out using micro-CT. As shown in **Figure 2A**, YGPs prominently reduced DMM-induced osteophyte formation. The parameters including BV/TV, Tb. Th and Tb. Sp of each group are shown in **Figures 2B–D**. The results showed that, following the DMM surgery, all the parameters for the model group were significantly different compared to sham group (*P* < 0.001). After YGPs treatment, the BV/TV and Tb. Th measurements of YGPs group were significantly lower than those of DMM-induced mice (*P* = 0.005, *P* < 0.001). Conversely, the Tb. Sp measurements of the YGPs group were significantly higher than those of DMM-induced mice (*P* = 0.027). These results suggest that YGPs have a positive effect on bone health in DMM-induced mice.

### DMM-Induced Downregulation of pSMAD-2 Is Enhanced by YGPs

**FIGURE 3:**
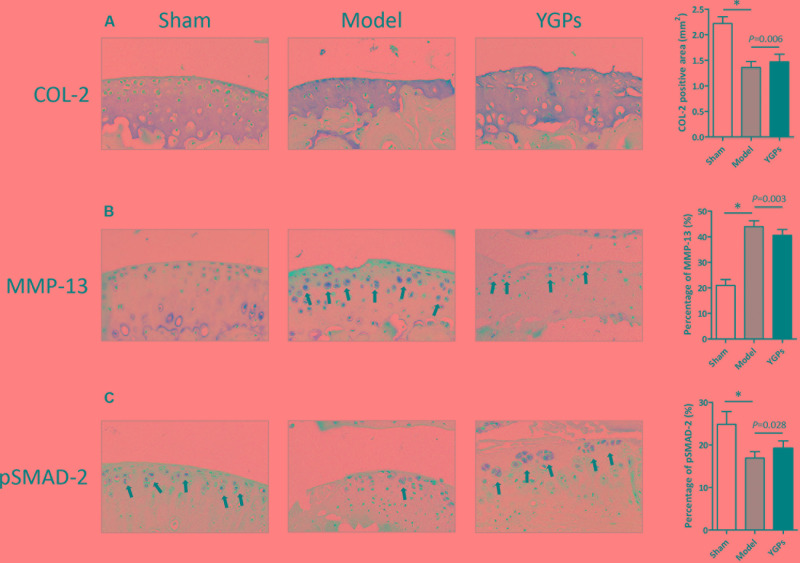
Yougui pills reduce MMP-13 and elevate pSMAD-2 expressional levels in articular cartilage of mice following DMM. Representative sagittal sections image (200 × ) **(A)** COL-2, **(B)** MMP-13 and **(C)** pSMAD-2 stained chondrocytes (brown; black arrows) with cell nuclei counterstained with hematoxylin (blue). Bars represent Mean ± SD (*n* = 10). ^∗^*P* < 0.001.

An important link in cartilage damage is the unbalance of synthesis and degradation of the ECM, an event marked by the expression of catabolic enymes such as MMP-13, and the key molecular of TGF-β/Smad signaling, pSMAD-2. Accordingly, it is possible that the chondroprotective effect on YGPs exhibited in **Figure 1** is associated with TGF-β/Smad signaling. To address this question, immunohistochemistry was conducted to evaluate COL-2, MMP-13 and pSMAD-2 protein expressional levels in the articular cartilage. As expected, DMM-induced mice had reduced levels of both COL-2 and pSMAD-2, and elevated levels of MMP-13 in cartilage compared to sham mice, indicating the suppression of TGF-β/Smad signaling (all, *P* < 0.001) (**Figures 3A–C**). Mice treated with YGPs were protected from the DMM induced COL-2 and MMP-13 positive staining in cartilage (*P* = 0.006, *P* = 0.003), suggesting that the maintenance of cartilage architecture in YGPs group could be due to inhibition of matrix degradation (**Figures 3A,B**). Interestingly, pSMAD-2 positive cells were increased in YGPs group (*P* = 0.028) (**Figure 3C**), indicating that YGPs may have been from cartilage degradation in part due to reduced MMP-13 production by chondrocytes residing in cartilage via activation of TGF-β/Smad signaling.

### YGPs Could Not Decelerate OA Progression in *TGF-βRII^Col2ER^* Mice

**FIGURE 4:**
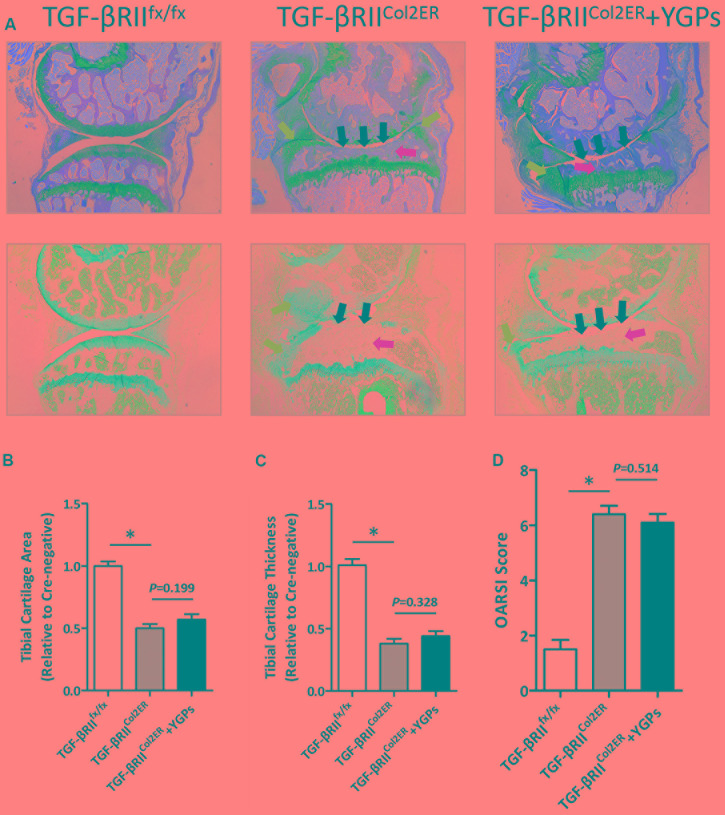
Yougui pills could not inhibit cartilage degradation in TGF-βRII^Col2ER^ mice. **(A)** Histological knee joint sections (50×) stained using Alcian Blue Hematoxylin/Orange G and Toluidine Blue. Joint degenerations are labeled (black arrows: cartilage degradation, blue arrows: osteophyte formation, yellow arrows: subchondral sclerosis). Cartilage architecture was evaluated using the Osteomeasure System to detemine the tibial cartilage area **(B)** and tibial cartilage thickness **(C)** OARSI scoring of the sections analyzed by histomorphometry was also performed **(D)** Bars represent Mean ± SD (*n* = 10). ^∗^*P* < 0.001.

The marked preservation of ECM produced by chondrocytes post-DMM in YGPs treated mice begged for subsequent investigation of the impact of YGPs on OA progression in *TGF-βRII^Col2ER^* mice. Firstly, we performed Alcian Blue Hematoxylin/Orange G and Toluidine Blue staining respectively to further explore the role of TGF-β/Smad signaling in the chondroprotective action of YGPs. Comparison of the sections from all groups revealed a severity of articular cartilage damage, subchondral sclerosis and osteophyte formation in *TGF-βRII^Col2ER^* mice. It should be noted that there were no discernable effects of YGPs treatment on inhibition of OA progression in *TGF-βRII^Col2ER^* mice (**Figure 4A**). Additional analyses were also performed using the histomorphometry approach as well as standardized OARSI cartilage scoring. Consistent with the histopathologic analysis, reduction of tibial cartilage area and thickness in *TGF-βRII^Col2ER^* mice were not mitigated significantly by YGPs treatment (*P* = 0.199, *P* = 0.328) (**Figures 4B,C**). Finally, the impact of YGPs on the articular cartilage in *TGF-βRII^Col2ER^* mice was also no significant difference based on OARSI scoring (*P* = 0.514) (**Figure 4D**). As analysis performed above, it should be noted that there were no significant differences in *TGF-βRII^Col2ER^* mice with or without YGPs treatment including tibial cartilage area and thickness, and OARSI cartilage scoring.

**FIGURE 5:**
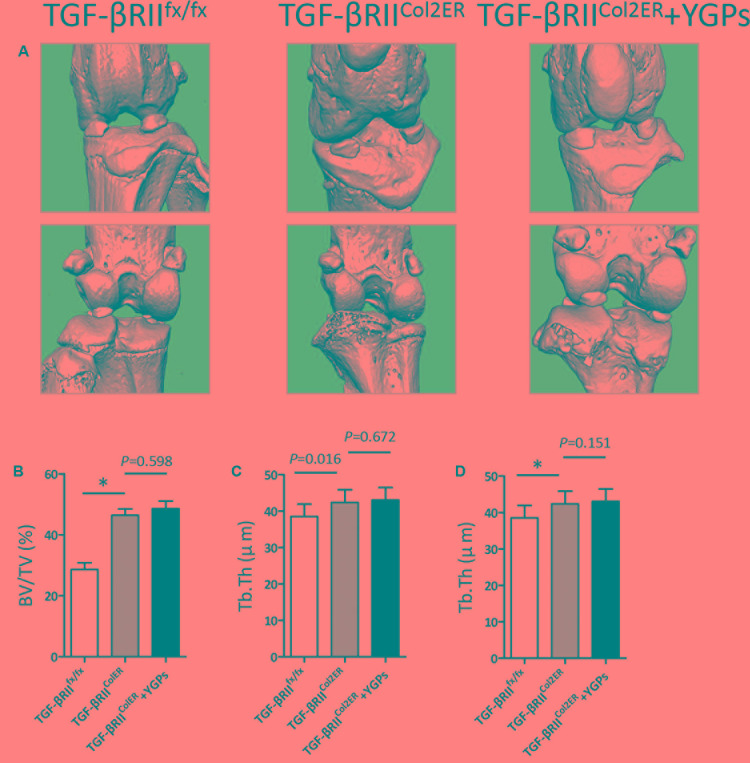
Changes in bone structure in TGF-βRII^Col2ER^ mice. **(A)** Representative micro-CT images. Quantification of the **(B)** BV/TV, **(C)** Tb. Th and **(D)** Tb. Sp by static histomorphometry. Bars represent Mean ± SD (*n* = 10). ^∗^*P* < 0.001.

Moreover, our micro-CT results also showed that YGPs almost can not inhibit the osteophyte formation (**Figure 5A**). In addition, BV/TV and Tb. Th were increased, and Tb. Sp was decreased in *TGF-βRII^Col2ER^* mice, whereas no significant improvement was observed in the animals treated with YGPs (**Figures 5B–D**).

### Upregulation of MMP-13 in *TGF-βRII^Col2ER^* Mice Is Not Ameliorated by YGPs

**FIGURE 6:**
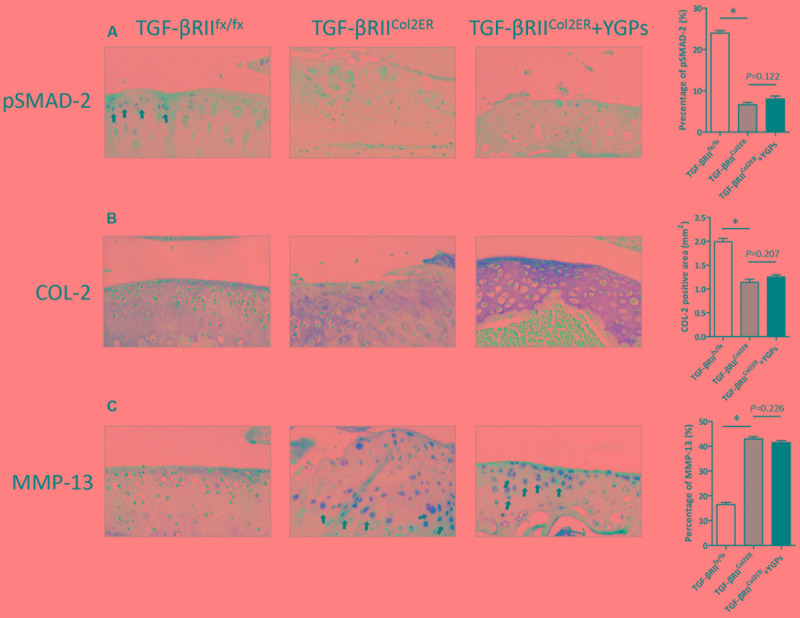
Upregulation of MMP-13 in TGF-βRII^Col2ER^ mice is not ameliorated by YGPs. Representative sagittal sections image (200×) **(A)** pSMAD-2, **(B)** COL-2 and **(C)** MMP-13 stained chondrocytes (brown; black arrows) with cell nuclei counterstained with hematoxylin (blue). Bars represent Mean ± SD (*n* = 10). ^∗^*P* < 0.001.

To understand better the role of TGF-β signaling in the chondroprotective action of YGPs, we further performed immunohistochemistry to evaluate COL-2 and MMP-13 protein expressional levels based on *TGF-βRII* gene deletion in chondrocyte. As shown in **Figure 6A**, *TGF-βRII^Col2ER^* mice had reduced levels of pSMAD-2 more than 80% in the cartilage compared to *TGF-βRII^fx/fx^* mice, suggesting that TGF-β/Smad signaling was almost blocked in our study. Interestingly, *TGF-βRII^Col2ER^* mice treated with YGPs were not protected from the COL-2 positive staining in the cartilage (*P* = 0.207) (**Figure 6B**). Accordingly, MMP-13 positive cells were not reduced in *TGF-βRII^Col2ER^* mice treated with YGPs (*P* = 0.226) (**Figure 6C**), indicating that YGPs almost had no effect on inhibition of matrix degradation with suppressing of TGF-β/Smad signaling. To summaries, no differences were observed in the articular cartilage, as chondrocytes were all actively producing similar levels of both COL-2 and MMP-13 in *TGF-βRII^Col2ER^* mice with or without YGPs treatment (**Figures 6B,C**).

### Routine Blood Test

**FIGURE 7:**
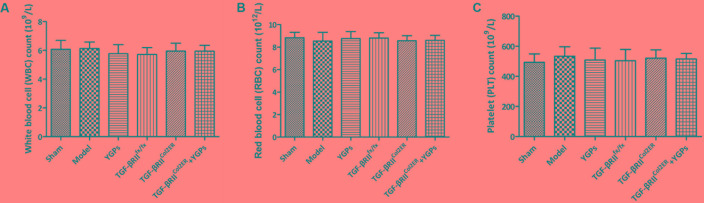
Hematological values in DMM-induced and TGF-βRII^Col2ER^ mice treated with YGPs. Hematological analysis was performed, including **(A)** White blood cell (WBC) count, **(B)** Red blood cell (RBC) count and **(C)** Platelet (PLT) count. Bars represent Mean ± SD (*n* = 10).

To evaluate the effect of YGPs orally administered on hematologic values, routine blood test was performed at the end of the experiment, including WBC count, RBC count and platelet (PLT) count. As shown in **Figures 7A–C**, there were no significant differences in both healthy and YGPs treated mice. YGPs treatment did not cause a significant change in any of the hematologic parameters compared to the control groups.

## Discussion

In present study, experiments were performed to investigate the impact of YGPs on the joint degenerative process in a mouse model of DMM. Relative examination of cartilage architecture, area and thickness change revealed prominently chondroprotective effects in degenerative knee joints of OA. These new findings support the hypothesis that orally administration of YGPs is articular cartilage protective and disease modifying in osteoarthritic knee joints. Although the precise mechanisms have yet to be defined, significant increases in TGF-β signaling appear to be involved.

A number of previous researches have described TCM as being effective in inhibition articular cartilage degeneration in animal models of OA disease. For example, Zhu et al. described protective activity for cartilage via reducing expressions of MMP-3, ADAMTS-4 and elevating TIMP-1, TIMP-3 with the TCM Sanmiao formulae which was administered to rat model induced by potassium oxonate and cold bath (Zhu et al., 2016). Yuan et al. (2011) reported similar benefits with Juanbi capsules in rabbits subjected to plaster cast fixation. *In vitro* models, Monotropein exhibited anti-apoptotic and anti-catabolic effects on rat OA chondrocytes treated by IL-β (Wang et al., 2014). Thus, our findings are in keeping with these previous studies and extend the benefits of TCM to include the more commonly employed, DMM-induced model of OA. According to TCM theory, TCM formulae may regulate diseases on the basis of its rich medicinal materials. The constituents of YGPs were separated and defined by HPLC analysis, and 10 components were determined as major active material basis. Additionally, no obvious abnormality on the hematologic parameters appeared in our study, including WBC, RBC and PLT (**Figures 7A–C**). This suggests that the extended use of YGPs for the prevention of OA dose not affect primary routine blood indexes.

Reproducing features of OA in animal is pivotal to have a better understanding of disease mechanism and to evaluate response to potential therapies. DMM surgical model is a frequently used chronic model of OA in mouse, and strongly associated with clinical process (Glasson et al., 2007; Iijima et al., 2014). The transaction of the medial menisco-tibial ligament alters the mechanical stability of the joint and accelerates cartilage erosion. DMM model has become a gold standard in the field (Culley et al., 2015). DMM surgery was applied to demonstrate the importance of the crucial aggrecan- and collagen-degenerating enzymes in articular cartilage damage in mice deficient in *Admats-5* (Glasson et al., 2005; Stanton et al., 2005) and *Mmp-13* (Little et al., 2009). As expected in our study, DMM-induced mice exhibited significant reduced protein level of COL-2 as well as elevated level of MMP-13 (**Figures 2A,B**), indicating that the model of OA with the destruction of articular cartilage dynamic balance by enhancing the catabolic activity of ECM proteins was established successfully.

An experimental protocol was performed in the present study that involved oral administration of YGPs to mice that were induced to develop OA through DMM surgery. The dose of YGPs (3.5 g/kg/day) was chosen in the present study, which was set to be the body weight equivalent to the human dose according to the drug instruction (Xu et al., 2002). Structural evaluation of the articular cartilage of sham and osteoarthritic knee joints using both histomorphometric and OARSI scoring methods revealed that mice administered YGPs were chondroprotected (**Figures 1B–D**). Tibial articular cartilage was preserved (**Figure 1A**), possibly via reduction of MMP-13 and elevated pSMAD-2, the key molecule of TGF-β/Smad signaling (**Figures 2B,C**).

The role of TGF-β signaling in the pathogenesis of OA has gained attention in recent years. In chondrocytes, TGF-β signaling is through specific membrane receptors (type I receptors also known as activin receptor like kinases, ALKs and type II receptors, TGF-βRII) and its intracellular effectors, the Smad proteins (van der Kraan et al., 2009). Inhibition of TGF-β/Smad signaling leads to increased damage to cartilage (Yang et al., 2001) and prevents osteophyte formation (Scharstuhl et al., 2003). To further explore whether the chondroprotection of YGPs in osteoarthritic mice is through TGF-β signaling, *Tgfbr2* was conditionally knocked out in the articular cartilage of mice at the age of 2 weeks in this study (Shen et al., 2013). As expected in the present study, severe articular cartilage damage was also observed in *TGF-βRII^Col2ER^* mice. While no overt chondroprotective effect was observed in articular cartilage of the *TGF-βRII^Col2ER^* mice supplemented with YGPs (**Figure 3A**). In addition, the lack of TGF-β/Smad signaling may activate runt-related transcription factor-2 (RUNX-2)-induced expression of MMP-13 (Chen et al., 2012). However, there were no discernable MMP-13 level changes of the supplements in *TGF-βRII^Col2ER^* mice (**Figure 4B**), suggesting a chondroprotective effect of YGPs may partially through TGF-β/Smad signaling in articular cartilage.

Overall, our data showed that YGPs are chondroprotective in OA through enhancing TGF-β/Smad signaling activity in articular cartilage, setting the stage for further mechanistic study and evaluation of joint structural modifications.

## Ethics Statement

All animal experiments were reviewed and approved by the Committee on the Ethics of Animal Experiments of Zhejiang Chinese Medical University and adhered to the guidelines of the Guide for the Care and Use of Laboratory Animals.

## Author Contributions

LZ and PW performed the main experiments of this work. JY, XJ, and CL contributed to the materials acquisition and data analysis. TX, SX, and RD conducted the animal experiment. HJ and PT designed this work. LX improved the design of this work. LZ wrote the paper. HJ critically reviewed all content. All authors approved the version of the manuscript.

## Conflict of Interest Statement

The authors declare that the research was conducted in the absence of any commercial or financial relationships that could be construed as a potential conflict of interest.
