# Supplementary material for: Yougui Pills Attenuate Cartilage Degeneration via Activation of TGF-β/Smad Signaling in Chondrocyte of Osteoarthritic Mouse Model
Source: Front Pharmacol. 2017 Sep 5;8:611. doi: 10.3389/fphar.2017.00611 (PMC5591843; doi:10.3389/fphar.2017.00611)
Supplement: Supplementary file 1 [file Image_1.PDF]

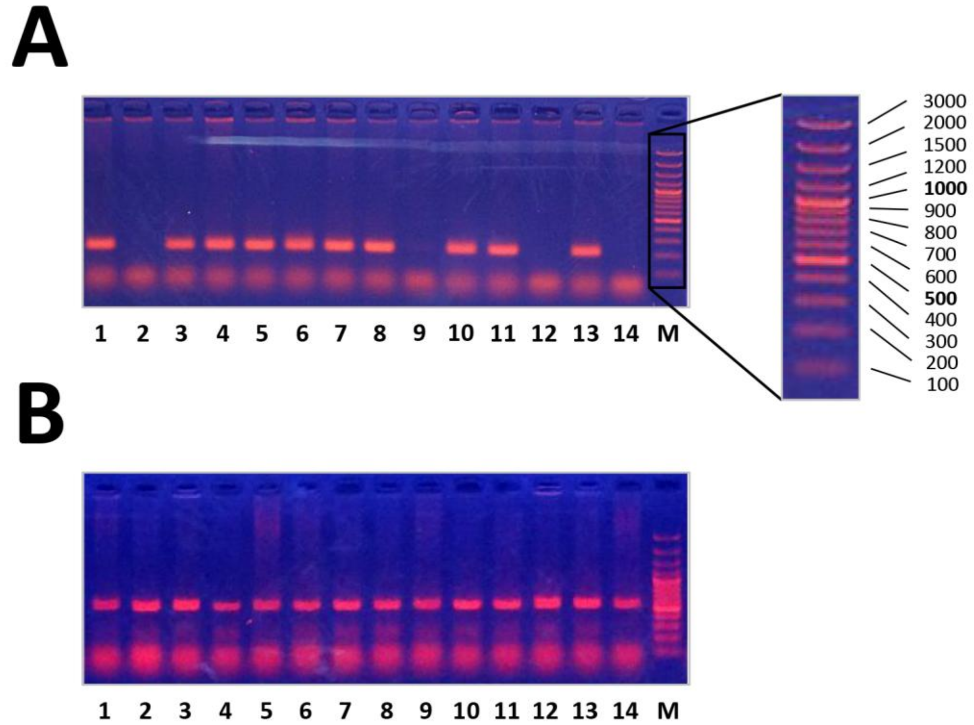

Supplemental Figure 1. Genotyping of $TGF\text{-}\beta RII^{Col2ER}$ and $TGF\text{-}\beta RII^{fx/fx}$ mice. **(A)** PCR amplification products of *Cre* in mice tail biopsy tissues. Line M is DNA size marker (between 3000 and 100 bp). Line 1, 3-8, 10, 11 and 13, positive samples. **(B)** PCR amplification products of *Tgfbr2* in the same mice tail biopsy tissues. Line M, DNA size marker (between 3000 and 100 bp); 1-14, positive samples. Based on the electrophoretic result showed in (A) and (B), Line 1, 3-8, 10, 11 and 13 are samples from $TGF\text{-}\beta RII^{Col2ER}$ mice; 2, 9, 12 and 14 are samples from $TGF\text{-}\beta RII^{fx/fx}$ mice.
